# Extramedullary and extranodal manifestations in chronic lymphocytic leukemia – an update

**DOI:** 10.1007/s00277-024-05854-1

**Published:** 2024-07-25

**Authors:** Tadeusz Robak, Anna Puła, Marcin Braun, Ewa Robak

**Affiliations:** 1 Department of Hematology, Medical University of Lodz, Ciołkowskiego 2, Lodz, 93-510 Poland; 2 Department of General Hematology, Copernicus Memorial Hospital, Lodz, Poland; 3 Department of Hematooncology, Copernicus Memorial Hospital, Lodz, Poland; 4 Department of Pathology, Chair of Oncology, Medical University of Lodz, Lodz, Poland; 5 Department of Dermatology, Medical University of Lodz, Lodz, Poland

**Keywords:** Chronic lymphocytic leukemia, Small lymphocytic lymphoma, Extranodal, Extramedullary, Infiltrations

## Abstract

Chronic lymphocytic leukemia/small lymphocytic lymphoma (CLL/SLL) is a common leukemia characterized by clonal expansion of mature CD5+/CD23 + B cells in the blood, bone marrow (BM) and lymphoid tissues. CLL can undergo extramedullary and extranodal infiltration, with one study noting an incidence of only 0.3 per 100,000 people, and in 17.6% of CLL patients in another report. The most common extranodal sites of leukemic involvement are the skin and central nervous system; however, other organs, including liver, lungs, kidney, gastrointestinal tract, bone, prostate and heart, are occasionally involved. The prognostic significance of extra-medullary CLL is still under debate, but the prognosis in such patients seems to be better in the era of novel targeted drugs. Following a diagnosis of extranodal CLL, survival appears to depend on the site of infiltration. This review presents an overview of CLL in patients with extramedullary and extranodal leukemic lesions, focusing on its epidemiology, pathogenesis, prognosis, clinical characteristics and treatment results.

## Introduction

Chronic lymphocytic leukemia/small lymphocytic lymphoma (CLL/SLL) is the most common form of adult leukemia in the Western world, characterized by monoclonal B cell proliferation and accumulation of mature lymphocytes within various organs including the peripheral blood (PB), bone marrow (BM), lymph nodes and spleen [1, 2]. CLL can be divided into *typical* and *atypical* forms. A diagnosis of typical CLL requires 5000 or more clonal B-lymphocytes/µL in PB and the coexistence of CD19, CD20, CD5, and CD23, and the restriction of light chain immunoglobulin. However, atypical CLL is morphologically and immunophenotypically distinct from typical CLL [3]. Immunophenotypically, atypical CLL is characterized by lack of expression of one or fewer surface antigens, most commonly CD5 and CD23, and it does not meet the criteria for diagnosis of any other B-cell lymphomas. An incidence of 5.1 cases per 100,000 individuals has been reported [4]; however, CLL is rarer in Asian populations, with an incidence rate 0.05 to 03/100,000 per year [5, 6]. Even so, it is twice as likely to manifest in men than in women. In the United States, an estimated 20,380 new cases, including 12,130 male and 6610 female, and 4490 deaths were reported for 2023 [7]. Most CLL patients are older, with a median age of 72 years at diagnosis [2].

The clinical course of CLL/SLL is variable. Most of the patients are asymptomatic at diagnosis with only a minority presenting with clinical symptoms; these include constitutional symptoms, a rapid increase of lymphocytes in PB and symptomatic lymphoid tissue enlargement [1]. Approximately, 2–10% of patients with CLL/SLL develop an aggressive lymphoma, known as Richter’s syndrome (RS) or Richter’s transformation (RT) [8].

CLL primarily involves the lymphatic system, but other non-lymphatic organs also demonstrate some degree of infiltration by circulating leukemic cells. A frequency of 0.3 per 100,000 people per year has been reported in some studies [9], and 17.6% of CLL patients in others [9]. Extramedullary and extranodal manifestations in CLL/SLL can occur with or without the presence of systemic CLL, and most often involve the skin (33%) and central nervous system (CNS) (27%) [10]. However, various other organs such as the liver, lungs, gastrointestinal tract, bone, prostate and heart are also occasionally involved in CLL, as well as BM and lymphoid tissue (Table 1) [10–15].

**Table 1:** Extranodal involvement in chronic lymphocytic leukemia

Involved organ	Freqency	Clinical characteristics	Diagnostic procedures	Prognosis	Treatment	References
Skin	3–5% of CLL patients	Solitary, grouped, or generalized papules, plaques, nodules, or large tumors.	Skin biopsy specimens with immunohistochemical staining	Not affected by CLL skin involvement, unfavorable in the RS transformation	Immunochemotherapy usually not effective, more effective venetoclax and BTK inhibitors	21,24–28,36, 37
Central nervous system	Symptomatic CNS involvement 0.4 − 2%, autopsy – 71%	Headaches, convulsions, ataxia, facial paralysis, cerebellar signs, visual abnormalities, motor and/or sensory deficits	Imaging studies and lumbar puncture	OS from CLL diagnosis 3.8 years, and average OS from CNS development 12 months	BTK inhibitors and venetoclax, penetrate the BBB and should be useful in patients with CLL and CNS involvement	38–41, 43, 44–46,50, 51,53
Cardiac involvement	CLL/SLL and other lymphomas in the 1.3% of all primary cardiac neoplasms	Mostly asymptomatic, pericardial invasion, constrictive pericarditis, endocardial fibroelastosis, congestive heart failure, coronary occlusion	Multimodality cardiac imaging, including echocardiography and cardiac MRI, CT scan and PET imaging	CLL infiltration – median 37.5 m; RT – median 4 m	No recommendations available, Drugs active in CLL	62–64, 68,69, 73, 74
Pulmonary infiltrations	5% of all extramedullary CLL, autopsy – 41% infiltrates in the lungs	Dry cough, progressive dyspnea, chest pain, and hemoptysis	HRCT, CT imaging -consolidations, centrilobular nodules, ground-glass opacities, transbronchial biopsy and bronchoalveolar lavage	62% patients died at median 41 months	No data available for CLL, poor in RT	76–82
Gastrointestinal tract	Real frequency not clear, microscopic infiltration in 50% of cases at autopsy	Usually asymptomatic, hemorrhage, gastric or colon perforation, intussusception, chronic diarrhea	In endoscopy polypoid lesions, hemorrhage, erosions, ulcers.	Good prognosis in most patients with CLL, poor prognosis in GI RT	Proper treatment of CLL improved GI symptoms.	86–95
Liver involvement	Abnormal LFTs at CLL diagnosis − 5%, 25% develop liver dysfunction within 10 years	Liver enlargement, and mild elevations in LFT, rare bile duct damage or liver failure	LFT, radiographic imaging, PET, liver biopsy, indications for liver biopsy: liver lesion, abnormal LFT, hepatosplenomegaly	Liver involvement correlate with advanced clinical stage and OS	Proper CLL treatment (immunochemotherapy, targeted drugs) can reverse liver failure and resolution of symptoms	95–104
Kidney involvement	Renal insufficiency in 7.5% at diagnosis, kidney infiltration in autopsy up to 90%	Increased kidney size, renal insufficiency	Imaging stydy, via ultrasound, MRI or CT scan, kidney biopsy	Median OS in patients with CLL kidney infiltration 84 m (range, 12–206 m)	Preferred BTK inhibitors rather than venetoclax in CLL patients with symptomatic kidneys involvement	108–118
Prostate and bladder infiltration	At autopsy of 88 cases persistent prostatic infiltration with CLL, in 18 (20,4%)	Mostly asymptomatic, obstructive urinary symptoms, dysuria and hematuria.	USG, CT. transrectal ultrasound (TRUS), prostate biopsy, cystoscopy	No data available	Targeted drugs, radiation therapy in prostatic involvement and local symptoms	119–126
Bone involvement	23 cases reported	Bone lesions in imaging investigation, hypercalcemia	CT, FDGPET / CT	Poorer outcome with associated hypercalcemia	Ibrutinib effective for osteolytic lesions, rare bone cortex reconstruction	127–133

Abbreviations: BBB - blood–brain barrier, BM – bone marrow, CR – complete response, FDG – fluorodeoxyglucose, HRCT - high-resolution computed tomography; LFT – liver function tests, LN – lymph nodes, MRI – magnetic resonance imaging, NHL – non-Hodgkin lymphoma, NR – no improvement, OS - overall survival, FDGPET/CT - fluorodeoxyglucose-positron emission tomography, /CT, PR – partial response, VCP – vincristine, cytarabine and prednisone, VMP – vinblastine, methotrexate, prednisone.

The approaches to treating CLL have undergone significant changes in recent years. While previous regimens favored chemo-immunotherapy (CIT), typically with fludarabine, cyclophosphamide and rituximab (FCR), bendamustine and rituximab (BR) or chlorambucil combined with rituximab or obinutuzumab [16, 17], modern treatment has been revolutionized with the use of targeted drugs, including Bruton tyrosine kinase (BTK) inhibitors (ibrutinib, acalabrutinib, zanubrutinib), phosphoinositide 3-kinases (PI3k) inhibitors and the B-cell lymphoma 2 (BCL-2) inhibitor venetoclax [18–20]. This article presents an overview of CLL characterized by extramedullary and extranodal leukemic involvement and its epidemiology, pathogenesis, prognosis, clinical characteristics and treatment results.

## Skin infiltration

**Fig. 1:**
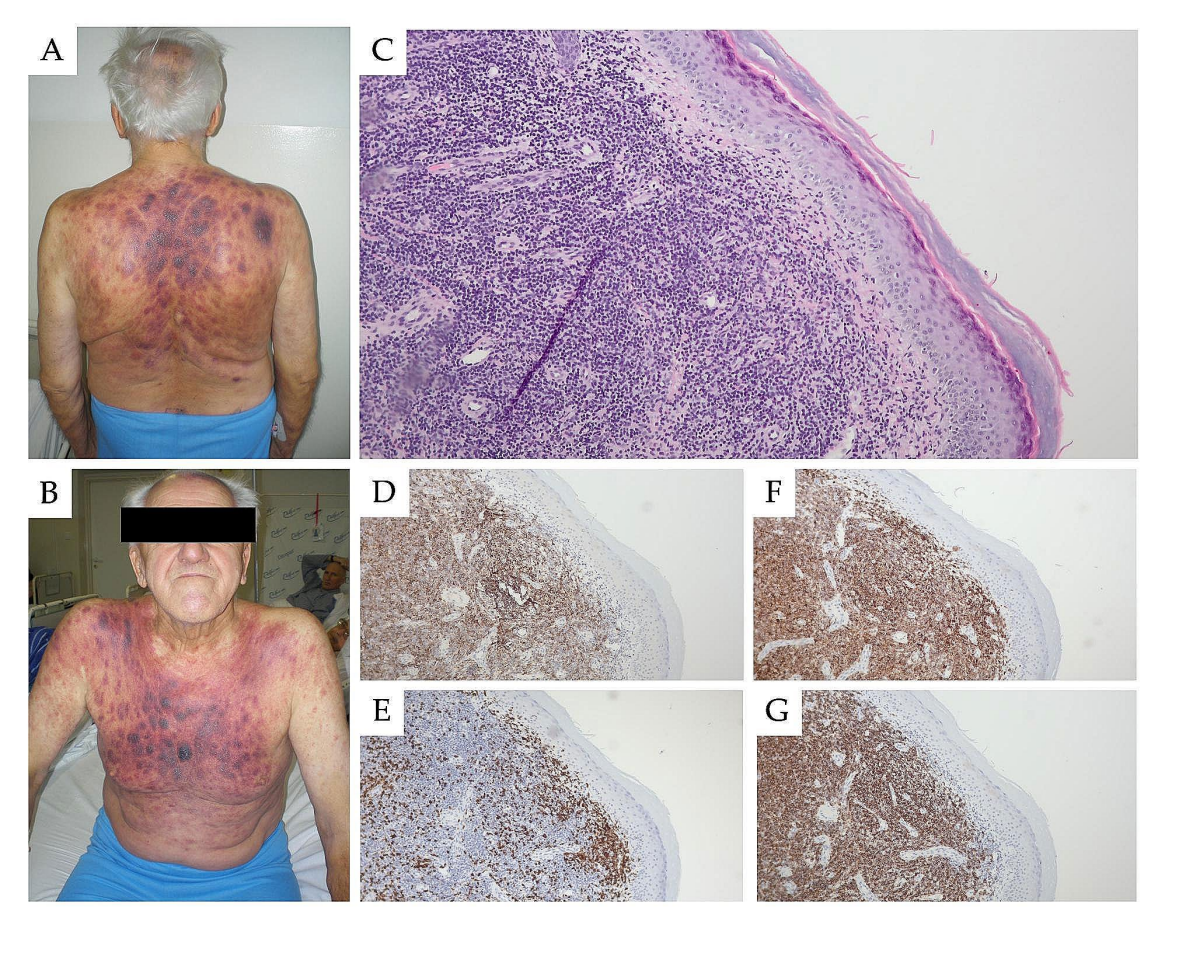
Skin changes in a 75 year old patient with CLL; they are present in the form of symmetrical nodular infiltrates and numerous, scattered red-blue papules and nodules with a hemorrhagic reaction on the torso (**A** and **B**). Biopsy of the skin torso infiltration in hematoxylin and eosin staining (**C**) showed strong positivity in immunohistochemistry (magnification 100x) for CD23 (**D**), negativity for CD3 (**E**), and strong positivity for CD5 (**F**) and CD20 (**G**)

In rare cases, i.e. in fewer than 5% [21], patients with CLL can demonstrate leukemic skin involvement known as leukemia cutis (LC) [21–23]. Skin lesions should be differentiated from cutaneous melanoma and non-melanoma skin cancers, which still occur more commonly in patients with CLL [24]. Thiesen et al. diagnosed CLL/SLL histologically in 3% of CLL cases with skin manifestations [22]. However, a systematic search for English language articles published between 2000 and 2019 identified only 56 CLL cases with leukemic skin lesions [25]. Cutaneous changes are usually diagnosed at an advanced stage of the disease, and rarely represent the initial manifestation. When present, skin infiltration can manifest as solitary, grouped, or generalized papules, plaques, nodules, or large tumors (Fig. 1). The most common skin site manifestations are the head and neck (33.9% of lesions), and trunk or extremities (26.8%) [25–29].

Cerroni et al. analyzed various clinical, histopathologic, immunophenotypic, and molecular features from 42 patients with CLL demonstrating specific cutaneous infiltrates [27]. The mean duration of CLL before skin manifestations was 39 months (range 0 to 142 months), and skin lesions were the first sign of CLL in seven patients (16.7%). Follow-up data could be obtained from 31 patients. The two patients with RS died after five and eight months. The 5-year survival of the patients was 66.6%.

**Fig. 2:**
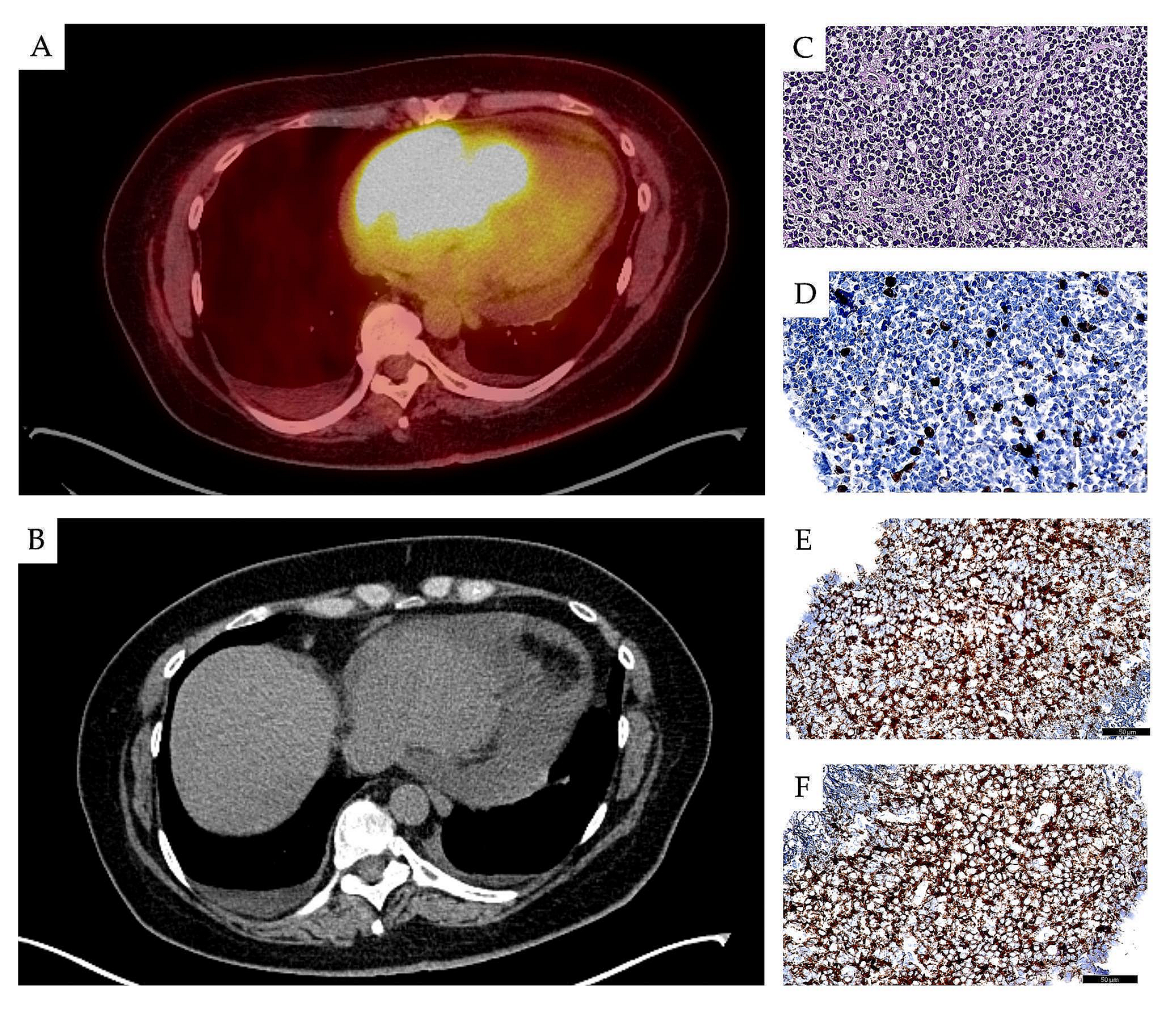
A PET/CT (**A**) and a CT scan of a CLL/SLL heart infiltration. Infiltration of CLL/SLL in intracardiac biopsy (digitalized, Philips IntelliSite scanner) of the masses in right ventricular epicardium with cytomorphology of small B-cells (**C**) immunophenotype showed negativity for CD3 (**D**) and strong positivity for CD23 (**E**) and CD5 (**F**)

A diagnosis of LC requires evaluation of skin biopsy specimens with immunohistochemical staining confirming the expression of characteristic cell surface markers; this also allows the case to be differentiated from reactive, inflammatory, and infectious skin lesions (Fig. 2) [26, 30]. Nodular or diffuse infiltrations with CLL cells in the dermis and/or subcutaneous tissue are most commonly observed; however, the leukemic infiltrations usually do not involve the epidermis or upper dermis. Cutaneous paraneoplastic disorders are more common than leukemic infiltration. Non leukemic skin changes are observed in 40% of patients with CLL: these include petechiae leukocytoclastic vasculitis, and neutrophilic dermatoses such as pyoderma gangrenosum and Sweet’s syndrome [31]. Moreover, disseminated skin infections, including candidiasis and herpes zoster have also been reported [32, 33].

Although the prognosis of CLL is usually not affected by skin involvement [26, 27, 34], it can be unfavorable in the event of RS transformation of CLL with specific infiltration of the skin [28, 34, 35]. Unfortunately, no consensus currently exists regarding the treatment of patients with LC in CLL. Until recently, the most common options were local radiotherapy, chemotherapy alone, and immunochemotherapy [25]; however, immunochemotherapy with chlorambucil and rituximab, BR or FCR is only rarely effective. Fortunately, more recent approaches based on targeted treatment with venetoclax and BTK inhibitors have been found to be more effective, and complete response (CR), including elimination of all skin changes, can be achieved [36, 37].

## Central nervous system involvement

Symptomatic CNS involvement with CLL cells has been observed in 0.4% to as many as 2% of cases [38–41]. However, a large autopsy study by Barcos et al. suggests that CNS involvement may be underdiagnosed in CLL patients, with CNS involvement detected in 20% of cases and leptomeningeal involvement in 8% [42]. Elsewhere, postmortem analyses identified extramedullary spread of CLL to the CNS in as many as 71% of cases [43]. A study performed in the Mayo clinic found the unmutated immunoglobulin heavy chain (*IGHV*) gene to be associated with a higher risk of CNS involvement (67% vs. 33%, *p* = 0.04) [38].

Patients with leukemic CNS involvement present with diverse and unspecific symptoms, including headaches, convulsions, diplopia, ataxia, facial paralysis, cognitive dysfunction cranial nerve palsies, cerebellar signs, visual abnormalities, and motor and/or sensory deficits [44–46]. However, imaging studies are not specific and sensitive and the diagnosis is usually supported by lumbar puncture [45, 47, 48].

The discovery of monoclonal B-cells in the cerebrospinal fluid (CSF) should prompt differentiation between contamination with CLL cells from peripheral blood, the transient presence of leukemic cells due to increased permeability of the blood-brain barrier (BBB) caused by inflammatory or infectious disease, and clinically significant central nervous system (CNS) involvement by CLL. However, flow cytometry analysis only offers 42% specificity on CSF for diagnosis of symptomatic CLL involvement in CNS from other diseases [38].

The risk factors for CNS involvement in CLL have not been established and cases of CNS infiltration do not seem to share any obvious common feature [43, 49]. In some patients, CNS involvement develops in the early stage of disease and is not associated with any high-risk chromosomal abnormality, such as del17p or del11q. A study of 78 cases of CLL with CNS involvement found that mean age of the patient to be 63.4 years, and the mean latency between CLL diagnosis and first signs of CNS involvement to be 2.6 years [50]. Mean overall survival (OS) from CLL diagnosis was 3.8 years, and mean OS from CNS development only 12 months. A more recent study of 50 patients found a mean latency 4.9 years [46].

The optimal treatment of patients with CLL and CNS involvement has not been established, and no standard protocol exists. Previously, intrathecal chemotherapy with methotrexate, cytarabine, and corticosteroids used alone or in combination showed promise in achieving CSF clearance. However, despite the initial benefit, disease relapse and eventual progression were later reported [51]. Novel targeted drugs, particularly BTK inhibitors and venetoclax, penetrate the BBB and should be useful in patients with CLL and CNS involvement [46, 52]. Ibrutinib was effective in the treatment of CNS lesions of mantle-cell lymphoma (MCL) and Waldenström macroglobulinemia [53, 54]. Some recent reports indicate that these drugs are also effective in CLL with CNS infiltration [46, 55, 56]. Venetoclax, a selective inhibitor of BCL-2, also penetrates the CSF and may be effective in such cases [56, 57]. Venetoclax can be combined with high-dose methotrexate and rituximab with good efficacy in some patients [58]. At present, the combination of venetoclax with ibrutinib seems to be the best option for these patients [57, 59, 60].

## Cardiac involvement

Cardiac manifestation of CLL/SLL, or other lymphomas, is an exceedingly rare event [15], representing around only 1.3% of all primary cardiac neoplasms [61–63]. Moreover, most CLL/SLL patients with heart infiltration are asymptomatic.

Pericardial involvement by CLL/SLL cells, constrictive pericarditis and leukemic infiltration in the myocardium and endocardium have been reported in CLL/SLL patients (Fig. 2) [15, 64–66], as well as leukemic involvement of the mitral or aortic valve and myocardium, endocardial fibroelastosis and intractable congestive heart failure, and CLL infiltration in the atrium and ventricle [67, 68]. Cardiac infiltration and coronary occlusion has also been reported [69–71]. Most of the reported cases with heart involvement had no evidence of RT at the time of heart symptom manifestation.

A recent literature search identified 18 well-described cases with CLL/SLL cardiac infiltration [15]. In three patients, cardiac manifestation was diagnosed before CLL/SLL diagnosis, while in the others, CLL/SLL in the heart was diagnosed between five months and 20 years from CLL/SLL diagnosis. Eight patients presented with pericardial invasion and constrictive pericarditis. Another two demonstrated infiltration of the coronary vessels by CLL/SLL cells, leading to acute myocardial infarction and sudden death due to coronary attack, with infiltration of the coronary vessels by CLL/SLL cells. Three cases presented with CLL infiltration in the heart valves and were diagnosed before cardiac symptoms. Cardiac CLL requires multimodality cardiac imaging, including echocardiography and cardiac MRI, CT scan and PET imaging (Fig. 2) [72].

Patients with CLL and cardiac infiltration demonstrate variable degrees of survival. Gordon et al. demonstrated a median survival of 37.5 months, which was considerably longer than the median four months observed in patients with aggressive B-cell lymphoma [62]. No recommendations are available on how to treat patients with CLL/SLL and cardiac involvement. Management of these patients depends on their previous history and clinical characteristics of heart infiltration. In some patients, cardiac symptoms have been found to improve following treatment with antileukemic drugs [15, 72, 73].

## Pulmonary infiltrations

Respiratory symptoms are frequently identified in patients with CLL. However, they are most commonly attributed to infections or lymphadenopathy, and leukemic pulmonary infiltrations (LPI) are rare [74]. The lung presentation has been reported in about 5% of extramedullary CLL. However, autopsy studies indicate the presence of extramedullary involvement in over 90% of patients, with 41% exhibiting leukemia infiltrates in the lungs [42]. The symptoms of pulmonary leukemic infiltrates are usually nonspecific, and include dry cough, progressive dyspnea, chest pain, and hemoptysis. The optimal approach for recognizing CLL with lung involvement is high-resolution computed tomography (HRCT) imaging. CT imaging commonly identifies the following in patients with pulmonary infiltrates of CLL: consolidations, centrilobular nodules with a tree-in-bud pattern, ground-glass opacities and interlobular septal thickening, as well as irregular thickening of the bronchovascular bundle and prominent peripheral pulmonary arteries [75–78]. However, these findings are non-specific and should be differentiated with atypical pneumonia, mycobacterium infection, and hypersensitivity pneumonitis.

Leukemic lung infiltration can be diagnosed by transbronchial biopsy and bronchoalveolar lavage [79]. Hill et al. analyzed 49 lung biopsies from 38 patients with CLL/SLL. LPI was found in two of 21 cases (9.5%) with acute inflammation, and in seven of 18 cases (38.8%) without any acute or chronic inflammation [80]. However, LPI was not found among 10 cases with chronic inflammation. The study indicates that LPI is uncommon in CLL patients with concurrent inflammation, and that LPI usually represents a specific leukemic process not related to inflammation. The findings also indicate that bronchoscopy with transbronchial biopsy is an effective technique for investigating whether pulmonary biopsies of lymphocytic infiltrations present a dense CLL infiltrate, which is typically distributed around the bronchial and vascular tissues [80]. Leukemic infiltrations exhibit prominent lymphangitic and vasocentric dispersal, affecting minor arteries, arterioles, alveolar septal capillaries and venules. In some patients, endobronchial CLL infiltrations causing bronchial obstruction and subsequent atelectasis, have also been reported; in addition, granulomas in CLL, occasionally with necrotic characteristics, can be observed, unrelated to any discernible infection [78, 79, 81]. Pulmonary involvement typically develops several years after diagnosis of CLL: Carmier et al. reported six patients with symptomatic pulmonary infiltrates of CLL which preceded pulmonary infiltration by three to fourteen years [81].

More recently, Tzilas et al. identified 13 patients with CLL and LPI at Mayo clinic from 1998 to 2022; the LPI was confirmed by lung biopsy [82]. All patients presented CLL with a median duration of 96 months (range: 50–408) and all were previously untreated. The most common pulmonary symptoms included dyspnea (62%) and cough (54%), and two patients (15%) were asymptomatic. CT imaging found intrathoracic lymphadenopathy to be present in all patients. In addition, most common symptoms included single or multiple nodular/mass-like opacities, documented in 10 patients (77%). Moreover diffuse centrilobular nodules, pleural mass, and diffuse bronchial wall thickening were observed, each in one patient. After diagnosis of LPI, antileukemic treatment was initiated in seven patients (54%), with six patients (86%) responding. Eight patients (62%) were found to have died at a median follow-up of 41 months. Incidentally, direct pulmonary infiltrates are regarded as an initial manifestation of CLL [77]. Bronchial obstruction due to massive endobronchial leukemic infiltrates has also been reported in patients with RS [83, 84].

## Gastrointestinal tract involvement

**Fig. 3:**
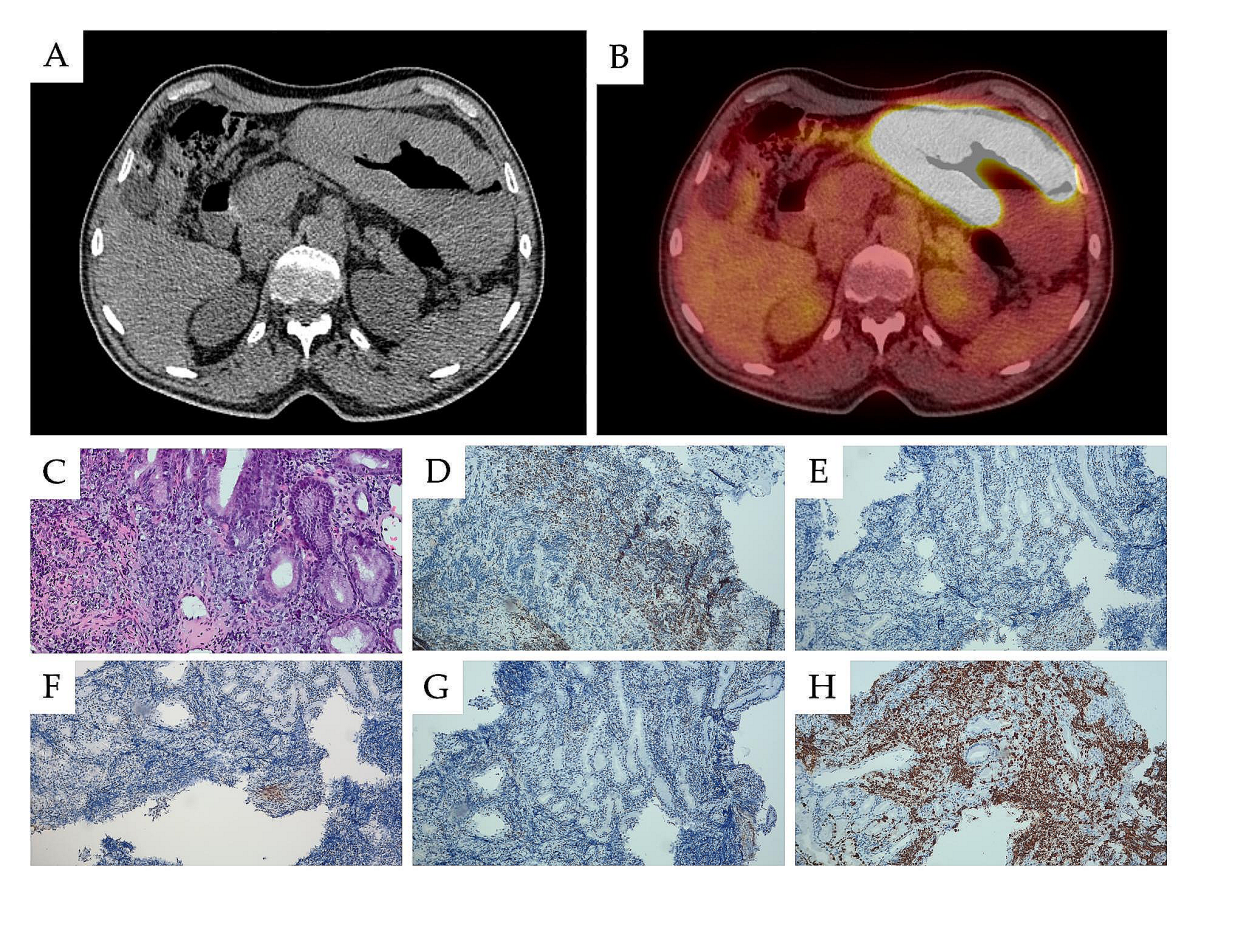
Richter transformation in the stomach visible in a CT (**A**) and a PET/CT scan (**B**). A biopsy (magnification 100x) showing infiltration by diffuse large B-cell lymphoma in hematoxylin and eosin staining (**C**), weak positive in immunohistochemistry for CD5 (**D**), while negative for BCL6 (**E**), CD10 (**F**) and CD23 (**G**), and showing high proliferation index Ki-67 of about 85% (**H**)

Gastrointestinal involvement (GI) of CLL is rarely clinically symptomatic [85–87]. At endoscopy, CLL is more commonly seen in the stomach, ileum, and proximal colon, and rarely so in the distal colon. The majority of CLL cases with GI involvement occur in the context of RT (Fig. 3) [88, 89]. However, in post-mortem studies, the reported incidence of microscopic infiltration can be as high as 50% of cases. This complication is usually asymptomatic. Some patients have reported chronic diarrhea as an initial presentation of CLL/SLL [85, 90]. GI involvement of CLL can also manifests as hemorrhagic events in the stomach, ileum, or proximal colon, or even gastric or colon perforation and intussusception [88–93].

In CLL, GI infiltration depends on tumor burden and proliferation activity. However, most patients with CLL do not receive endoscopic investigation, and the true frequency of gastrointestinal involvement remains unclear. It is recommended that patients exhibiting GI symptoms should receive endoscopies, including biopsies, to exclude or confirm possible gastrointestinal CLL manifestations [94]. In endoscopy, GI lesions manifest as polypoid lesions, hemorrhage, polypoid lesions, ulcers or ulcerated erosions. While suitable treatment of CLL improved GI symptoms in most patients with CLL and GI lesions, gastrointestinal RT generally carries a poor prognosis.

## Liver involvement

Liver infiltration manifests as liver enlargement, mild elevations in liver function tests (LFT) to acute liver failure [95]. These symptoms are mostly related to leukemic periportal infiltration. At the time of CLL diagnosis, about 5% of patients have abnormal LFTs, and approximately 25% develop liver dysfunction within 10 years [96]. The most common area of liver involvement is the portal tract, followed by sinusoidal, nodular, and extensive infiltration seen in RT [95]. Furthermore, nonspecific canalicular stasis, hepatomegaly, ascites and RT have been reported in rare cases [95, 97–99], and B-cell CLL also appears to have been associated with bile duct damage in some patients [97]. Some cases of atypical CLL presentation with acute liver failure secondary to CLL leukemic infiltration have also been reported [100, 101].

A diagnosis of leukemic hepatic infiltration requires a liver biopsy [97, 102]. However, the indications for liver biopsy have not been thoroughly described in the literature. Schwartz et al. report the presence of leukemic infiltrates in 98% of evaluated liver histology sections from 47 cases of CLL [103]. The pathological abnormalities included expansion of the portal tracts, bridging infiltration, bridging fibrosis, and cirrhosis with pseudo-lobule formation.

Hampel et al. reviewed the indications for biopsy, the description of the pathological findings, CLL therapy and outcomes among 52 patients with CLL/SLL from Mayo clinic who underwent liver biopsy [96, 104]. The most common indications for liver biopsy were liver lesion, identified on radiographic imaging in 21 (41%) patients, followed by abnormal liver function tests in 17 (33%) patients and hepatosplenomegaly in 11 (21%) patients. In three (6%) patients, the indication for liver biopsy was unknown. CLL/SLL involvement was identified in 38 of the 52 (73%) patients undergoing liver biopsy, including RS in four (8%) patients. Infiltration of the portal tracts by CLL/SLL was the most frequent localization of CLL infiltration. Only nine patients (24%) with leukemic infiltration received CLL treatment. Two patients obtained CR, two PR and one a stable disease. However, most patients (29/38; 76%) with CLL liver involvement did not receive immediate, CLL-specific treatment after diagnosis of liver infiltration [104].

Severe hepatic infiltration is commonly associated with high-grade transformation to RT. However, it is extremely rare for isolated RT to present as a solid liver tumor [101, 105]. Although PET/CT is very useful for establishing a correct diagnosis of RT, it should be confirmed by biopsy.

Although liver infiltration of CLL is a common event, liver failure is rare, excluding RT [96]. Nevertheless, liver involvement correlates with advanced clinical stage and shorter OS [65]. Confirmation of liver dysfunction at diagnosis does not influence time to first treatment; however, these patients tend to demonstrate shorter OS than those with normal LFTs [96]. While chemotherapy induced reversal of liver failure and prompt resolution of symptoms in some cases, it was ineffective in others [100].

## Kidney involvement

Renal insufficiency was observed in 7.5% of CLL patients at diagnosis, and up to 16.1% during follow-up [106]. However, leukemic involvement of the genito-urinary system, including the kidney, bladder and prostate, is rare [107, 108]. Ratterman et al. report that 10% of studied CLL patients had leukemic infiltration in the genitourinary and gynecological systems, including the kidneys [74]. However, in CLL, kidney biopsy is not routinely performed to explain renal insufficiency due to an indolent course of disease. The low rate of kidney biopsy is a limiting factor in better understanding CLL leukemic infiltration. Strati et al. report that only 1.2% of 4,024 patients with CLL in the Mayo clinic between 1995 and 2014 underwent kidney biopsy [109]. After a median follow-up of 60 months (range 12 to 216 months), 19 (39%) patients died; this group included one of ten (10%) patients with membranoproliferative glomerulonephritis (MPGN), four of six (66%) patients with CLL infiltration as the primary etiology of the renal failure, and two of six (33%) patients with thrombotic microangiopathy (TMA).

**Fig. 4:**
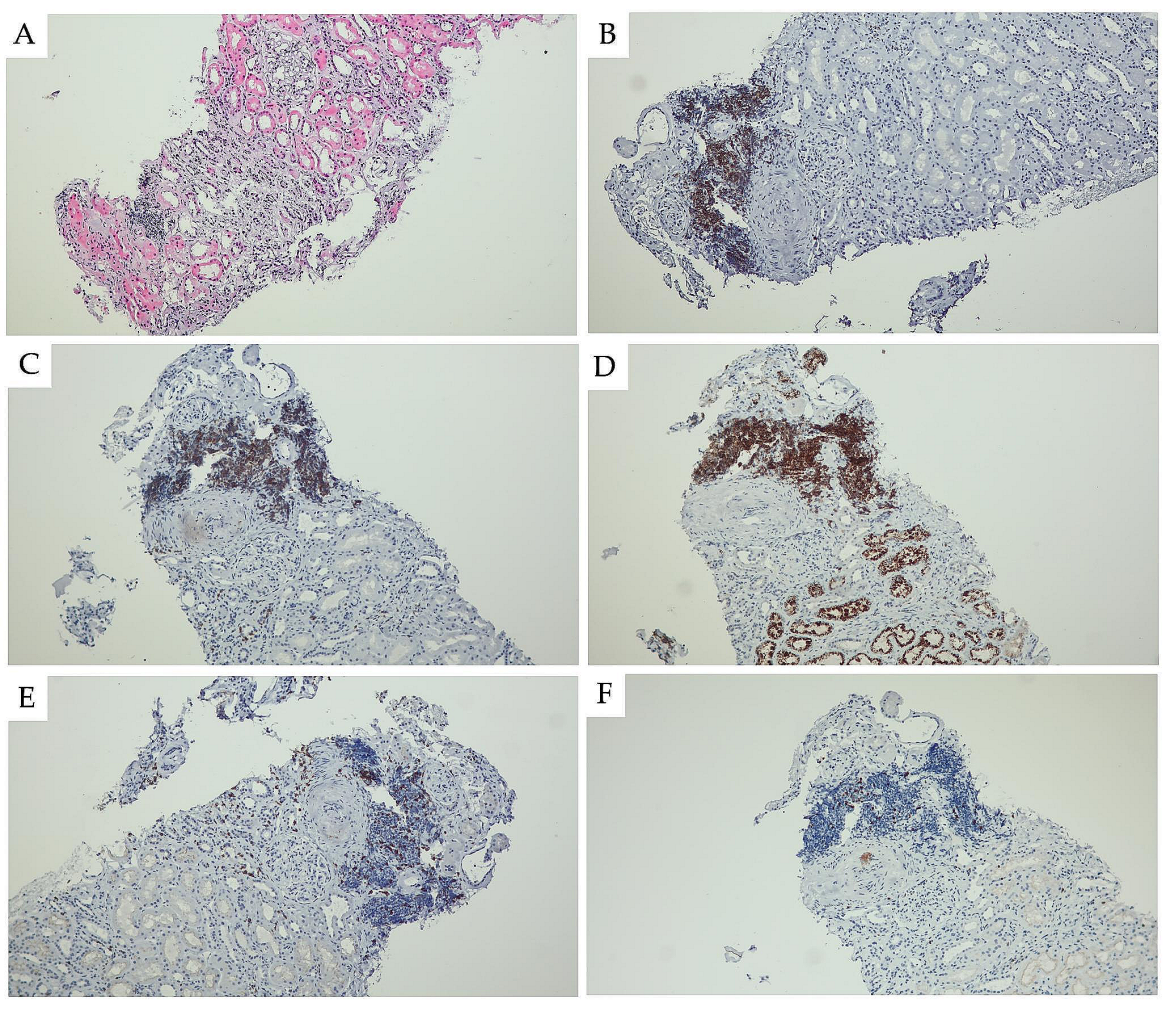
Infiltration of CLL/SLL in kidney biopsy (magnification 100x) with cytomorphology of small B-cells (**A**). Immunohistochemistry showed strong positivity for CD20 (**B**), CD5 (**C**), and CD23 (**D**); and negativity for CD3 (**E**). The proliferation index Ki-67 was low - of about 10-15%

Renal interstitial infiltration is more common in autopsy studies, ranging from 10 to 90% of CLL/SLL patients (Fig. 4) [110, 111]. Wang et al. identified 10 patients with CLL cell involvement in the renal interstitium [111]. The extent of the infiltrating CLL cells ranged from 10 to 90% of kidney parenchyma, and six (60%) patients had infiltrating CLL cells ≥ 50%. Moreover, three patients (30%) expressed monoclonal immunoglobulins in the infiltrating CLL cells. Diffuse infiltration with more than 50% CLL cells was associated with severe renal insufficiency. The authors suggest that CLL cells infiltrating the renal interstitium secrete monoclonal immunoglobulins, directly causing renal injury by secreting monoclonal immunoglobulins in situ. However, the mechanism of renal insufficiency in patients with kidney CLL is heterogenous and several factors may contribute to the pathogenetic mechanism of renal injury. Moreover, diffuse kidney infiltration by CLL cells can compress the renal tubules and microvasculature, thus increasing the risk of intrarenal obstruction and ischemia [109, 112].

Granulomatous interstitial nephritis (GIN) secondary to CLL was reported in five patients by Nasr et al. [113]. Patients presented with severe renal failure due to both GIN and leukemic interstitial infiltration. Most responded to steroids with or without anti leukemic chemotherapy. Treatment of CLL with concomitant renal abnormalities is difficult, mainly due to the increased toxicity of several drugs, especially purine analogs. Less nephrotoxic drugs, including a variety of alternative regimens, have been used to treat CLL-associated membranoproliferative glomerulonephritis, including prednisone, chlorambucil, rituximab and bendamustine either alone or in combination [109]. However, at the time of novel targeted therapies, kidney disease can also influence the treatment choice [114]. Nephrotoxicities are particularly associated with venetoclax combined with rituximab or obinutuzumab, mainly due to tumor lysis syndrome. From this reason, some authors recommend BTK inhibitors rather than venetoclax in CLL patients with symptomatic CLL involvement of the kidneys [11, 106]. However, the acute kidney injury has also been reported in CLL patients treated with ibrutinib. Long-term ibrutinib administration can also induce degenerative tubular damage [115]. On the other hand, ibrutinib was found to be effective in treating CLL-associated membranous nephropathy [116]. Strati et al. report the median OS in patients with CLL kidney infiltration to be 84 months (range, 12 to 206 months) (111).

## Prostate and bladder involvement

Prostate and bladder involvement by CLL cells has been observed occasionally (108, 109). In a postmortem study, Zein et al. report that 18 of 88 cases with CLL (20.4%) showed persistent prostatic infiltration by leukemic cells [117]. Other studies identified CLL infiltration in 0 to 0.8% of patients undergoing radical prostatectomy and lymph node dissection [118–120].

In the majority of reported cases, CLL infiltration was asymptomatic. However, some patients were symptomatic, including two recently-reported cases [121–123]. Thomas et al. reported a patient with CLL and who developed sequential prostatic and penis leukemic infiltration one year following the diagnosis of CLL. The patient was treated with ibrutinib and showed a symptomatic response shortly after treatment initiation; however, 14 months later, he was diagnosed with urinary bladder CLL infiltration, but responded to single-agent rituximab [122]. Elsewhere, D’Arena et al. present a patient with CLL who developed leukemic infiltration of the prostate and penis in the advanced phase of disease; the patient responded well to conventional treatments with chlorambucil followed by bendamustine [123]. However, radiation therapy is recommended as treatment of choice in patients with prostataic or penile involvement and local symptoms [123].

Only seven cases with extramedullary CLL involving the bladder have been reported so far [124]. All patients presented with dysuria and hematuria. Cystoscopy with biopsy is important for proper diagnosis. Treatment with chemotherapy or transurethral resection are successful in most patients. Moreover, venetoclax was used in one patient, resulting in improvement in leukocytosis and without any recurrence of hematuria [124].

## Bone involvement

Skeletal involvement in CLL is very rare, being more frequent in other lymphoproliferative disorders and acute leukemias. In a literature review, Bacchiari et al. identified axial skeleton or proximal long bone involvement in 11 of 22 cases with CLL and skeletal lesions [125]. Multiple fractures were reported, including the axial skeleton or proximal long bones, in eight patients. The fractures were localized to the skull or facial bones in three patients and to the skull or facial bones in rare cases. Importantly, in 13 patients, osteolysis or pathological fracture was the first symptom at CLL diagnosis.

Another study analyzed nine CLL patients compromised by osteolytic bone lesions with no evidence of Richter’s syndrome [126]. Five of the patients had co-existing hypercalcemia. Bone involvement by lymphoma cells, with resistant hypercalcemia, has been also reported as the first manifestation of RT [127]. The presence of osteolytic bone lesions with hypercalcemia in CLL/SLL is associated with a poorer outcome, with a poor response to chemotherapy or immunochemotherapy [128, 129]. However, novel targeted drugs can be more effective in CLL patients with bone involvement. Some studies indicate that ibrutinib was effective for treating osteolytic lesions of CLL, including a bone cortex reconstruction [129–131].

## Conclusions

Extramedullary and extranodal organ involvement in CLL is exceedingly rare and may be a manifestation of advanced stage of the disease. However, the true incidence of extranodal infiltration by CLL cells is difficult to estabish. The most common sites of organ involvement include the skin, CNS and gastrointestinal tract. Patients with CLL and unexplained organ dysfunction should receive a prompt evaluation in case of leukemic infiltration. The prognostic significance of extramedullary CLL is unknown. Survival after diagnosis of extramedullary and extranodal CLL appears to depend on the site of extramedullary involvement. Symptomatic extranodal CLL is an indication for initiation of antileukemic therapy; in these instances, treatment should take into consideration the site of disease and the potential drug-induced compromise of organ function.

## Data Availability

No datasets were generated or analysed during the current study.
